# Gardner P, Snyderman C, Jankowitz B. Vascular Challenges in Skull Base Surgery. Thieme, New York, USA, 2022, 242 pages. 410 illustrations. ISBN 978-1-68420-068-9

**DOI:** 10.3325/cmj.2022.63.498

**Published:** 2022-10

**Field of medicine:** Neurosurgery, otorhinolaryngology, neurology, medical education

**Audience:** Neurosurgeons, otorhinolaryngologists, neurologists, senior residents engaged in skull base surgery, and other interested physicians

**Purpose:** The book provides a well-written, comprehensive overview of the vascular challenges in skull base surgery. It details management strategies and algorithms regarding skull base and endoscopic endonasal approaches, surgical techniques, related pathologies, and potential complications. In each chapter, numerous illustrations allow a detailed representation of surgical anatomy. The content is presented through text, tables, graphs, and neuroradiological, cadaveric, and intraoperative pictures. The book provides a wealth of information and can serve as a useful resource for all interested readers.

**Content:** The content is divided into 22 well-organized chapters, each focused on a specific topic.

The first chapter, *Vascular Anatomy of the Head and the Neck/Circle of Willis,* briefly reviews the important anatomical concepts of the head and neck vasculature and their relation to skull base and cerebrovascular surgery. The head and neck circulatory system is presented as anterior and posterior circulation, with a review of the major branches, common variants, and their clinical significance.

The second chapter, *Evaluation of Tumor-Involved Vasculature (Including Balloon Test Occlusion),* briefly discusses arterial and venous anatomy of the skull base and imaging techniques for the skull base vascular tumors. Here, the authors propose balloon test occlusion as a method to adequately and safely resect a vascular base tumor.

The third chapter, *Embolization of Skull Base Tumors,* reviews different methods for embolizing skull base tumors including common pitfalls and perils with a few case examples, management strategies, and complications.

The fourth chapter, *Vascular Supply of Local-Regional Flaps in Skull Base Surgery,* briefly describes the reconstruction of skull base defects using extranasal and endonasal reconstructive flaps. Different flaps are described in tables, and various figure examples are given.

The fifth chapter, *Bypass in the Treatment of Skull Base Tumors,* describes vascular challenges and ways to avoid injury when performing bypass, as well as management strategy, technical considerations, outcomes, and complications. Various case examples and alternative strategies are presented.

The sixth chapter, *Alternatives to Standard Bypass Techniques for Skull Base Tumors (Including Direct Imax Bypass),* briefly describes indications, the operative technique, and the alternative technique to traditional bypass in the context of skull base tumors. A novel method using the internal maxillary artery as donor vessel is presented, with an overview of its indications, operative method, graft choices, and potential problems.

The seventh chapter, *Skull Base Approaches for Aneurysms,* details the application of approaches to the skull base for intracranial aneurysm surgery, ie, transzygomatic, transpetrosal, fat lateral suboccipital, and transcondylar approach. The chapter also lists vascular challenges and related pathologies, as well as provides case examples with description of management strategies and potential complications.

The eighth chapter, *Endoscopic Endonasal Aneurysm Treatment,* reviews the current application of endoscopic endonasal surgery for intracranial aneurysms. The chapter gives the technical details when it comes to managing these aneurysms and provides various case examples with description of management strategies and algorithms, as well as potential complications.

The ninth chapter, *Dealing with Major Intraoperative Vascular Injury during Endonasal Approaches to the Anterior Skull Base,* reviews the relevant anatomy of endoscopic endonasal approach to the anterior skull base, the common vascular complications, and the methods to minimize these complications. Case examples with management algorithm and potential complications are also provided.

The tenth chapter, *Dealing with Major Intraoperative Vascular Injury,* reviews the key elements in understanding vascular and endoscopic challenges, preoperative planning, landmarks, rescue steps, as well as postoperative management and complication avoidance in the setting of major vascular injury. Case examples with management algorithms and strategies are also provided.

The eleventh chapter, *Dealing with Major Intraoperative Vascular Injuries During Endonasal Posterior Fossa Surgery,* provides a brief overview of the prevention and surgical management of major vascular injuries during endoscopic endonasal posterior fossa surgery. Illustrative cases with management analysis are also given.

The twelfth chapter, *Vascular Challenges in Anterior Skull Base Open Surgery,* provides an overview of relevant surgical anatomy of the anterior skull base. It also lists common anterior and anterolateral skull base approaches, pathologies involving the anterior cranial base vasculature, relevant vascular challenges, common vascular complications, and microsurgical techniques to avoid such complications.

The thirteenth chapter, *Dealing with Vascular Injury During Middle Fossa Surgery,* briefly describes vascular control and avoidance of injury and related pathologies of the middle cranial fossa. Case examples with management strategies and potential complications are also described.

The fourteenth chapter, *Posterior Fossa during Open Skull Base Surgery,* reviews the relevant and normal anatomy of the posterior fossa to help surgeons avoid major intraoperative injuries. It also discusses techniques for controlling bleeding and for the management of consequences.

The fifteenth chapter, *Perforator Injury During Endoscopic Endonasal Skull Base Surgery,* describes the anatomy of the perforators in a way that is logical for endoscopic skull base surgeons; different ways to prevent their injuries; and measures to take when injuries happen. Clinical examples are also discussed.

The sixteenth chapter, *Perforator Injury during Open Skull Base Surgery,* briefly discusses the vascular challenges with a detailed overview of basal perforator vessels derived from major cerebral arteries. It also lists the techniques to avoid perforator injury and related pathologies. Case examples with management algorithm are described.

The seventeenth chapter, *Endovascular Options to Treat Iatrogenic Vascular Injury and Tumor Involvement of the Skull Base,* describes the endovascular treatment options for acute and delayed vascular injury after skull base surgery; the utility and interpretation of balloon test occlusion; preoperative stenting for arterial protection during skull base tumor resection; and treatment strategies for concomitant internal carotid artery aneurysms and skull base tumors.

The eighteenth chapter, *Extracranial Anterior Cranial Base Surgery for Vascular Tumors,* focuses on angiofibromas as a good model for discussing the principles of management of all vascular tumors. It details preoperative assessment of tumor vascularity, tumor staging based on vascularity, preoperative devascularization, surgical strategy including the role of endoscopy, hemostatic surgical techniques, and prevention of complications.

The nineteenth chapter, *Extracranial Lateral Cranial Base Vascular Tumor Surgery,* briefly describes the pathophysiology, anatomy, diagnostic workup, and intraoperative management of the extracranial vascular lesions of the lateral skull base, focusing on the preoperative assessment of tumors involving the petrous internal carotid artery.

The twentieth chapter, *Venous Considerations in Skull Base Surgery,* gives details of venous anatomy, alternative approaches, and dissection and preservation of the veins. Injury to venous sinuses with a number of figure examples is also discussed.

The twenty-first chapter, *Neurophysiologic Monitoring and Its Role during Cerebrovascular Injury,* reviews the principles, technical aspects, interpretation, and limitations of each modality of intraoperative neurophysiologic monitoring, such as somatosensory, brainstem auditory, or transcranial motor-evoked potentials and EEG.

The twenty-second chapter, *Simulation and Training – Preparing for Vascular Injury,* provides overview of different surgical trainings such as simulation, including vascular injury simulation, cadaveric models, as well as different techniques that can improve the patients’ outcome.

**Highlights:** The book contains all essential information about skull base surgery and provides an excellent overview of the topic and all accompanied challenges. In almost every chapter, relevant anatomy is reviewed through numerous schemes and cadaver images. In addition, a number of skull base approaches, as well as endoscopic endonasal approach for anterior, middle, and posterior cranial fossa with special considerations are described, as well as potential vascular injuries. Specific conditions regarding endovascular treatment, intraoperative neurophysiologic monitoring, and surgeon training are discussed in separate chapters. The comprehensive index at the end of the book enables quick orientation and selective reading. The text is accompanied by color figures, schematic presentations, and neuroradiological images, which make the learning both easy and exciting.

**Related reading**: A number of other comprehensive medical references either in book or pocket-size handbook are available, such as: Vasculature of the Brain and Cranial Base, Thieme, 2015; Atlas of 360 Degree Skull Base Surgery, Thieme, 2021; Skull Base Surgery: Strategies, Thieme, 2019; Endonasal Endoscopic Surgery of Skull Base Tumors: An Interdisciplinary Approach, Thieme, 2015; Handbook of Skull Base Surgery, Thieme, 2015; Surgery of the Skull Base, Elsevier, 2018; Atlas of Endoscopic Sinus and Skull Base Surgery, 2nd Edition, Elsevier, 2018.

